# Association Analysis of *ULK1* with Crohn's Disease in a New Zealand Population

**DOI:** 10.1155/2012/715309

**Published:** 2012-03-20

**Authors:** Angharad R. Morgan, Wen-Jiun Lam, Dug-Yeo Han, Alan G. Fraser, Lynnette R. Ferguson

**Affiliations:** ^1^Department of Nutrition, Faculty of Medical and Health Sciences, The University of Auckland, Grafton, Auckland 1142, New Zealand; ^2^Nutrigenomics New Zealand, New Zealand; ^3^Department of Medicine, Faculty of Medical and Health Sciences, The University of Auckland, Grafton, Auckland 1142, New Zealand

## Abstract

The gene *ULK1* is an excellent candidate for Crohn's disease (CD) due to its role in autophagy. A recent study provided evidence for the involvement of *ULK1* in the pathogenesis of CD (Henckaerts et al., 2011). We attempted to validate this association, using a candidate gene SNP study of *ULK1* in CD. We identified tagging SNPs and genotyped these SNPs using the Sequenom platform in a Caucasian New Zealand dataset consisting of 406 CD patients and 638 controls. In this sample, we were able to demonstrate an association between CD and several different *ULK1* SNPs and haplotypes. Phenotypic analysis showed an association with age of diagnosis 17–40 years and inflammatory behaviour. The findings of this study provide evidence to suggest that genetic variation in *ULK1* may play a role in interindividual differences in CD susceptibility and clinical outcome.

## 1. Introduction

Crohn's disease (CD) is a form of inflammatory bowel disease (IBD) characterized by chronic, relapsing gastrointestinal inflammation. It results from multiple genetic and multiple environmental risk factors, operating additively and interactively. In recent years, the search for genetic determinants of CD has changed dramatically with the introduction of the Genome Wide Association Study (GWAS) technology from which results have been excellent. As well as helping to identify multiple susceptibility loci involved in the genetic susceptibility to CD, GWAS has also provided evidence for the involvement of biological pathways such as autophagy. Autophagy is a well-conserved regulatory process by which protein and organelle turnover occurs in cells by autodigestion through lysosomal degradation. The pathway can interact with other vital processes such as programmed cell death, inflammation, and immune mechanisms. Autophagy has several roles in innate and adaptive immunity including pattern recognition receptor signalling, regulation of cell death, elimination of bacteria and viruses, and immune cell homeostasis [1–3]. Thus, it is thought that CD may result from a defective autophagy pathway causing an impaired antibacterial response and so an ineffective control of bacterial infection, dysbiosis of the intestinal microbiota, and chronic inflammation.

There are many genes in the autophagy pathway that have been previously associated with CD. They code for proteins involved in the detection of autophagic triggers (*IRGM* [4–6], *NOD2* [7–9], *VDR* [10], and *DAP1* [11, 12]), orchestrating autophagosome formation (*ATG16L1* [13, 14]), or autophagosomal maturation (*LRRK2* [15]). Mutations in these autophagy-related genes may lead to loss of autophagic function and the subsequent development of Crohn's disease.

A recent study investigating a number of autophagy genes for their involvement in CD has described a novel association between *Unc-51-like kinase-1* (*ULK1*) and CD [16]. *ULK1* is a serine/threonine protein kinase that plays a critical role in the initial stages of autophagy, although the exact molecular mechanism is unknown.

Here, we attempted to validate the association of *ULK1* with CD in a well-characterised case-control New Zealand dataset. We considered not only allele and genotype frequencies, but also the question as to whether genotype could predict phenotype since this is an essential tool in understanding disease behaviour and future treatment requirements [17].

## 2. Methods 

### 2.1. Samples

A total of 1044 subjects from New Zealand were included in the study: 406 CD patients and 638 controls. All participants self-reported European ancestry. 

Clinical records were analysed to confirm diagnosis, and IBD status was defined using standard diagnostic criteria [18]. Cases were phenotyped according to the Montreal Classification systems. Clinical characteristics of the CD patients are shown in Table 1. 

Participants consented to collection of peripheral blood or a buccal swab for DNA extraction and genotyping, and DNA was extracted from the blood/buccal samples using Qiagen DNA extraction kit and following the manufacturer's instructions. 

The study was conducted under ethical protocol MEC/04/12/011, authorised through the New Zealand Multi-Region Human Ethics Committee. All study subjects gave informed consent. 

### 2.2. SNP Selection

Tag SNPs in *ULK1* were selected using HapMap release 28 and the tagger functionality within Haploview with pairwise tagging to identify SNPs using an *r* ^2^ > 0.8 and a minor allele frequency >5%. As a result, nine tag SNPs were selected for genotyping: rs10902469, rs7133672, rs7953348, rs7488085, rs11616018, rs12303764, rs4964879, rs3088051, and rs3923716. 

### 2.3. Genotyping

Genotyping was performed with the MassARRAY and iPlex systems of the Sequenom genotyping platform (Sequenom, San Diego, CA), which uses the MALDI-TOF primer extension assay [19, 20], according to manufacturers' recommendations. 

Assays were optimized in 24 samples consisting of 20 reference Centre d'Etude du Polymorphisme Humain (CEPH) samples and 4 blanks. 

All sample plates contained cases, controls, blanks, CEPH, and duplicate samples. Quality control measures included independent double genotyping and, where available, comparison of our CEPH genotypes to those in the Hapmap database (http://www.hapmap.org/). 

### 2.4. Statistical Analysis

SNPs were tested for deviation from HWE in both cases and controls using a chi-square goodness-of-fit test. To determine if there were differences between cases and controls, allele frequencies for each SNP were analyzed using 2 × 2 chi-square tables. 

Genotype and phenotype associations were assessed by comparing allele frequencies between controls and patient subgroups defined using the clinical characteristics. These analyses were carried out using R (R: a language and environment for statistical computing, R Foundation for Statistical Computing, Vienna, Austria. ISBN 3–900051-07-0, URL http://www.R-project.org/) and SAS (V9.1 SAS Institute., Cary, NC, USA). 

To determine linkage disequilibrium (LD) between SNPs and to define haplotype blocks, we uploaded our data into Haploview [21]. Haplotype blocks were defined using the default algorithm, which uses confidence intervals [22]. Haplotype analysis was carried using HAPLO.SCORE in R to test for association of these haplotypes with CD. 

For all analyses we considered a *P* value less than 0.05 to indicate statistical significance. 

The false discovery rate (FDR) was used to correct for multiple testing [23, 24]. 

## 3. Results 

Two SNPs, rs7133672 and rs4964879, failed in the genotyping assay. The remaining seven SNPs were all genotyped successfully and were in Hardy-Weinberg equilibrium in both cases and controls. 

### 3.1. Association Analysis

From the seven genotyped SNPs, we saw association with CD for two SNPs. The G allele of SNP rs10902469 was more frequent (95.4%) in the cases compared to controls (92.5%), OR = 1.69, *P* = 0.0084. The T allele of SNP rs7488085 was more frequent (93.7%) in the cases compared to controls (91.1%), OR = 1.46, *P* = 0.030. These SNPs remain statistically significant if we correct for multiple testing using the false discovery rate. Genotype and allele counts/frequencies and *P* values for all genotyped SNPs are shown in Table 2. 

### 3.2. Phenotypic Analysis

The two SNPs that were associated with CD (rs10902469 and rs7488085) were both associated with age of diagnosis 17 to 40 years (OR = 1.90, *P* = 0.010 and OR = 1.53, *P* = 0.044) and inflammatory disease (OR = 2.63, *P* = 0.002 and OR = 1.79, *P* = 0.018). SNP rs10902469 was also associated with colonic disease (OR = 2.33, *P* = 0.025). SNP rs3088051 was associated with stricturing (OR = 1.45, *P* = 0.015) and ileal (OR = 1.34, *P* = 0.042) disease and bowel resection (OR = 1.58, *P* = 0.002). The other 4 SNPs did not demonstrate any associations with any subphenotypes. Full phenotype results are shown in Table 3. All of the significant findings remained significant after multiple testing correction, with the exception of the association of rs7488085 with age of diagnosis 17–40. 

### 3.3. Haplotypic Analysis

**Figure 1:**
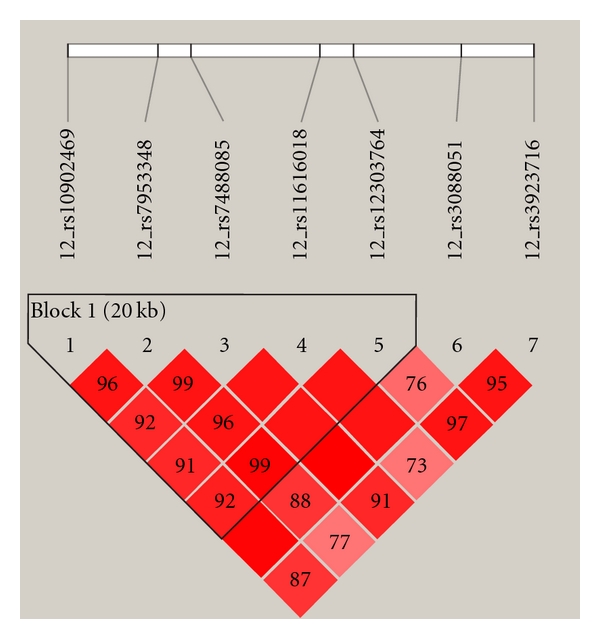
*ULK1* LD plot.

Figure 1 shows the LD plot for the *ULK1* SNPs in our New Zealand dataset. Five SNPs are in the same haplotype block: rs10902469, rs7953348, rs7488085, rs11616018, and rs12303764.  Table 4 summarises haplotype analysis results but in brief three haplotypes were found to be statistically significant in their association with CD. Haplotype CCCCT was protective in that it was more frequent in the controls (0.066) compared to cases (0.036), *P* = 0.005. Haplotype GCTTT was protective in that it was more frequent in the controls (0.019) compared to cases (0.006), *P* = 0.038. Haplotype GTTTT was more frequent in the cases (0.455) compared to controls (0.406), *P* = 0.027. However, after applying multiple testing correction these haplotypes were no longer statistically significant.

## 4. Discussion

*ULK1* is an autophagy gene that has recently been reported for the first time to be associated with CD [16]. In order to confirm the role of *ULK1* in CD susceptibility we performed an independent association study in a New Zealand case–control sample set. We were able to demonstrate evidence of association for two SNPs. However, the associations we observed were different from those reported by the previous study. Henckaerts et al. [16] had the strongest association with CD for rs12303764, but this SNP was not associated with CD in our samples. They also reported weaker associations for rs10902469, rs7953348, and rs3923716. From these we only saw association in our dataset for rs10902469. We also saw association with rs7488085, which was not genotyped in the previous study. To further determine whether *ULK1* is a CD susceptibility gene, we examined the data (data not shown) from a recent CD genome-wide meta-analysis [25] for SNPs in this gene. Three SNPs in *ULK1* were included in the analysis: rs11246867 that is LD (*r* ^2^ = 1) with rs10902469 that was associated with CD in both our study and the study by Henckaerts et al. showed no association in the GWAS meta-analysis, rs3923716 was associated with CD in the study of Henckaerts et al. but not in our study and likewise not in the GWAS meta-analysis, and rs3088051 was not associated with CD neither in the study of Henckaerts et al. nor our study (although there is a difference in cases and controls which is approaching statistical significance) but showed association in the GWAS meta-analysis (uncorrected nominal, *P* = 0.00068). It is by no means certain that support for *ULK1* as a CD susceptibility gene requires the same pattern of association to be obtained. Genetic heterogeneity, variation in phenotypes, and lack of power may explain some of the discrepancies. Further studies are needed in other cohorts to determine the robustness of these observations in different populations and to be certain whether *ULK1* can be described as CD susceptibility gene. 

Phenotypic analysis demonstrated association for the 2 CD associated SNPs with a young adult age at first diagnosis (17–40 years) and not with disease diagnosed after 40 years nor with early-onset (paediatric) disease (before 17 years). The age of diagnosis of CD in adults is known to have a bimodal distribution: the first peak occurs between the ages of 15 and 30 years, and the second peak occurs between the ages of 60 and 80 years [26]. Younger age-at-diagnosis patients represent a separate and often more severe phenotype of CD [27]. The different phenotypes seen in the different age groups are likely to be as a result of each of these groups having a different genetic component to their disease. The study we report here concludes that *ULK1 *has a role in patients who are diagnosed as young adults and is unlikely to be important in patients who are diagnosed after 40 years. Likewise there is no evidence to suggest *ULK1* has a role in paediatric CD, although this cannot be ruled out entirely as the numbers in this group are small and so the power to detect an association here is limited. 

Phenotypic analysis also demonstrated association with inflammatory CD behaviour. In terms of disease behavior inflammatory disease is the milder and less complicated form and over time some patients may develop penetrating or stricturing complications. So a strong association with inflammatory disease is difficult to interpret. But the association of *ULK1* with inflammation is not surprising. *ULK1* plays a critical role in the initial stages of autophagy and it is possible that genetic variation in this gene may result in autophagy-mediated control of commensal bacteria being compromised, subsequently leading to an intestinal inflammatory response to bacteria. 

The results from phenotype analysis also suggested that other subphenotypes may also be affected as SNP rs3088051 demonstrated association with stricturing behaviour, ileal location, and bowel resection. This SNP was not associated with CD in the main case-control analysis. However there was a difference in allele frequency between cases and controls that was approaching statistical significance. 

In conclusion, the findings of this study provide some evidence to suggest that genetic variation in *ULK1* may play a role in interindividual differences in CD susceptibility and clinical outcome. However it remains unclear which variants are most important. There could be other genetic variants such as rare variants and/or copy number variations that exist at this locus and are in LD with one or more of the SNPs we have investigated. It is known that the *ULK1* gene is located in a region of copy number variation [28, 29]. Future efforts should aim to identify the causative variants in this region by sequencing and functional experiments. 

## Figures and Tables

**Table 1:** Summary of clinical data of CD patients.

		CD
Gender	F	265 (65.6)
	M	139 (34.4)
Age at diagnosis	<17	46 (12.6)
	17 to 40	257 (70.2)
	40<	63 (17.2)
CD behaviour	Inflammatory	201 (55.1)
	Stricturing	118 (32.3)
	Penetrating	46 (12.6)
CD location	Ileal	136 (37.2)
	Colonic	119 (32.5)
	Ileocolonic	111 (30.3)
Bowel resection	N	270 (66.7)
	Y	135 (33.3)
Other IBD family	N	330 (89.7)
	Y	38 (10.3)
Perianal disease	N	329 (85.7)
	Y	55 (14.3)

**Table 2:** Genotype and allele counts (and frequencies) in CD patients and in controls.

SNP		Case	Control		Case	Control	OR (955 CI)	*P*
rs10902469	G/G	367 (90.8)	544 (0.86)	G	771 (95.4)	1177 (92.5)	**1.69 (1.14–2.50)**	0.0084*
	C/G	37 (9.2)	89 (0.14)	C	37 (4.6)	95 (7.5)		
	C/C	0 (0.00)	3 (0.00)					
rs7953348	T/T	276 (69.0)	406 (65.8)	T	663 (82.9)	993 (80.5)	1.17 (0.93–1.46)	0.17
	C/T	111 (27.8)	181 (29.3)	C	137 (17.1)	241 (19.5)		
	C/C	13 (3.3)	30 (4.9)					
rs7488085	T/T	355 (87.7)	529 (82.8)	T	759 (93.7)	1164 (91.1)	**1.46 (1.03–2.06)**	0.030*
	C/T	49 (12.1)	106 (16.6)	C	51 (6.3)	114 (8.9)		
	C/C	1 (0.2)	4 (0.6)					
rs11616018	T/T	281 (70.6)	419 (66.3)	T	668 (83.9)	1022 (80.9)	1.23 (0.98–1.55)	0.078
	C/T	106 (26.6)	184 (29.1)	C	128 (16.1)	242 (19.1)		
	C/C	11 (2.8)	29 (4.6)					
rs12303764	T/T	165 (40.8)	254 (40.3)	T	515 (63.7)	788 (62.5)	1.06 (0.88–1.26)	0.58
	G/T	185 (45.8)	280 (44.4)	G	293 (36.3)	472 (37.5)		
	G/G	54 (13.4)	96 (15.2)					
rs3088051	T/T	193 (47.7)	331 (52.2)	T	560 (69.1)	921 (72.6)	1.20 (0.99–1.46)	0.086
	C/T	174 (43.0)	259 (40.9)	C	250 (30.9)	347 (27.4)		
	C/C	38 (9.4)	44 (6.9)					
rs3923716	C/C	333 (82.4)	506 (79.9)	C	735 (91.0)	1134 (89.6)	1.17 (0.86–1.59)	0.30
	A/C	69 (17.1)	122 (19.3)	A	173 (9.0)	132 (10.4)		
	A/A	2 (0.5)	5 (0.8)					

*Remain statistically significant after applying a multiple testing correction using FDR.

**Table 3:** Phenotypic analysis results.

		rs10902469: G		rs7953348: T		rs7488085: T		rs11616018: T		rs12303764: T		rs3088051: C		rs3923716: C	
		OR (95% CI)	*P*	OR (95% CI)	*P*	OR (95% CI)	*P*	OR (95% CI)	*P*	OR (95% CI)	*P*	OR (95% CI)	*P*	OR (95% CI)	*P*
Age at diagnosis	<17	0.98 (0.44–2.18)	0.953	0.97 (0.58–1.64)	0.912	0.90 (0.44–1.85)	0.770	1.15 (0.66–2.01)	0.634	0.94 (0.61–1.43)	0.761	1.05 (0.65–1.70)	0.836	0.93 (0.46–1.86)	0.827
	17 to 40	**1.90 (1.17–3.10)**	0.010*	1.11 (0.86–1.44)	0.436	**1.53 (1.01–2.32)**	**0.044**	1.15 (0.88–1.50)	0.298	1.17 (0.95–1.45)	0.142	1.18 (0.94–1.48)	0.165	1.03 (0.73–1.46)	0.852
	40<	1.37 (0.62–3.04)	0.435	1.35 (0.82–2.22)	0.241	1.14 (0.57–2.25)	0.715	1.35 (0.81–2.26)	0.245	1.11 (0.77–1.61)	0.580	1.33 (0.90–1.97)	0.151	1.53 (0.75–3.11)	0.243
CD behaviour	Inflammatory	**2.63 (1.42–4.86)**	0.002*	1.25 (0.93–1.68)	0.139	**1.79 (1.10–2.91)**	0.018*	1.25 (0.93–1.69)	0.143	1.07 (0.85–1.35)	0.554	1.06 (0.82–1.36)	0.667	1.19 (0.80–1.76)	0.396
	Stricturing	1.19 (0.68–2.10)	0.547	1.00 (0.71–1.41)	0.994	1.19 (0.70–2.01)	0.516	1.17 (0.82–1.68)	0.392	1.20 (0.89–1.60)	0.229	**1.45 (1.08–1.95)**	0.015*	1.02 (0.64–1.63)	0.943
	Penetrating	0.84 (0.39–1.81)	0.658	0.97 (0.58–1.63)	0.912	0.72 (0.37–1.39)	0.323	0.95 (0.57–1.60)	0.850	1.29 (0.83–2.01)	0.265	1.17 (0.73–1.86)	0.509	0.85 (0.44–1.65)	0.633
CD location	Ileal	1.62 (0.89–2.94)	0.116	1.20 (0.85–1.69)	0.295	1.57 (0.91–2.70)	0.104	1.22 (0.87–1.73)	0.254	1.11 (0.85–1.45)	0.430	**1.34 (1.01–1.78)**	0.042*	1.15 (0.73–1.82)	0.541
	Colonic	**2.33 (1.11–4.89)**	0.025*	1.34 (0.92–1.95)	0.124	1.58 (0.88–2.81)	0.123	1.33 (0.91–1.95)	0.137	1.08 (0.82–1.44)	0.586	1.00 (0.73–1.37)	0.992	1.27 (0.77–2.09)	0.345
	Ileocolonic	1.19 (0.66–2.13)	0.565	0.89 (0.63–1.25)	0.499	0.99 (0.59–1.64)	0.954	1.01 (0.71–1.45)	0.951	1.19 (0.89–1.60)	0.245	1.21 (0.88–1.66)	0.237	0.86 (0.54–1.36)	0.517
Other IBD family	Y	6.10 (0.84–44.4)	0.075	1.01 (0.73–1.39)	0.968	3.67 (0.88–15.2)	0.073	1.40 (0.72–2.73)	0.327	1.08 (0.68–1.74)	0.737	1.24 (0.75–2.04)	0.412	2.07 (0.74–5.81)	0.169
Bowel resection	Y	1.12 (0.66–1.90)	0.674	0.99 (0.56–1.74)	0.963	1.09 (0.68–1.77)	0.717	1.22 (0.86–1.73)	0.270	1.18 (0.90–1.56)	0.225	**1.58 (1.19–2.10)**	0.002*	0.95 (0.61–1.46)	0.802
Perianal disease	Y	1.70 (0.67–4.29)	0.262	0.74 (0.48–1.16)	0.186	0.79 (0.42–1.50)	0.473	0.82 (0.52–1.30)	0.398	1.46 (0.96–2.23)	0.079	1.10 (0.71–1.71)	0.676	0.62 (0.36–1.09)	0.099

*Remain statistically significant after applying a multiple testing correction using FDR.

**Table 4:** ULK1 haplotype analysis.

rs10902469	rs7953348	rs7488085	rs11616018	rs12303764	Hap-score	*P*	Hap-frequency	
							Control	Case
C	C	C	C	T	−2.828	**0.005**	0.066	0.036
G	C	T	T	T	−2.071	**0.038**	0.019	0.006
G	T	T	T	G	0.011	0.991	0.361	0.363
G	C	C	C	T	0.151	0.880	0.019	0.020
G	C	T	C	T	0.333	0.739	0.094	0.098
G	T	T	T	T	2.212	**0.027**	0.406	0.455
